# Secondary Orbital Interactions Enhance the Reactivity of Alkynes in Diels–Alder Cycloadditions

**DOI:** 10.1021/jacs.8b13088

**Published:** 2019-01-29

**Authors:** Brian J. Levandowski, Dennis Svatunek, Barbara Sohr, Hannes Mikula, K. N. Houk

**Affiliations:** † Department of Chemistry and Biochemistry, University of California, Los Angeles, Los Angeles, California 90095, United States; ‡ Institute of Applied Synthetic Chemistry, TU Wien, 1110 Vienna, Austria

## Abstract

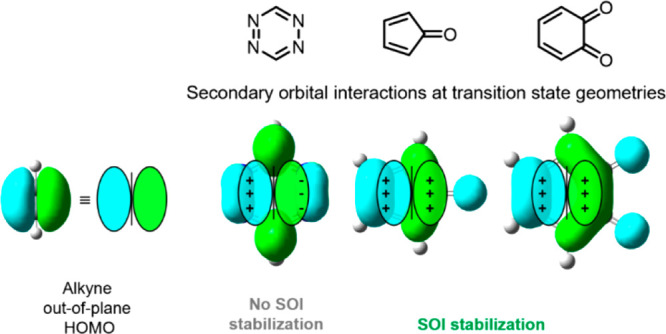

We have investigated the inverse electron-demand Diels–Alder reactions of *trans*-cyclooctene (TCO) and *endo*-bicyclo[6.1.0]nonyne (BCN) with a 1,2,4,5-tetrazine, a cyclopentadienone, and an *ortho*-benzoquinone. Tetrazines react significantly faster with TCO compared to BCN because the highest occupied molecular orbital (HOMO) of TCO is significantly higher in energy than the HOMO of BCN and there is less distortion of the tetrazine. Despite the different HOMO energies, TCO and BCN have similar reactivities toward cyclopentadienones, while BCN is significantly more reactive than TCO in the cycloaddition with *ortho*-benzoquinone. We find that the higher reactivity of BCN compared to TCO with *ortho*-benzoquinone is due to secondary orbital interactions of the BCN HOMO-1 with the diene LUMO.

The Diels–Alder (DA) reaction is a powerful synthetic tool that generates six-membered rings with remarkable regioselectivity and stereoselectivity.^1^ Using Frontier Molecular Orbital (FMO) theory, generalizations about the shapes and energies of the highest occupied (HOMO) and lowest unoccupied (LUMO) molecular orbitals can be applied to understand the reactivity, regioselectivity, and stereoselectivity of Diels–Alder reactions.^2^ Distortion energies are an additional factor that play an important role in DA cycloadditions.^3,4^ For example, cyclopentadiene and cycloheptadiene have similar FMO shapes and energies, but significantly different reactivities. The reactivities of these cyclic dienes are related to the energy required to geometrically deform the diene into the transition state geometry.^5^

Recently, Diels–Alder reactions have attracted attention as a tool for *in vitro* and *in vivo* labeling.^6,7^ These cycloadditions are bioorthogonal and require highly reactive and selective dienes and dienophiles that do not cross-react with biological nucleophiles. Few reactions satisfy these criteria, and the development of new bioorthogonal reactions is an active area of research.^8−10^Scheme 1 shows the experimental second-order rate constants for the inverse electron-demand Diels–Alder reactions of the bioorthogonal dienes 3,6-di-2-pyridyl-1,2,4,5-tetrazine (**1**), new experimental results reported here for a naphthalene-fused cyclopentadienone (**2**_**Ethyl**_), and a *t*-butyl substituted *ortho*-benzoquinone (**3**), with the bioorthogonal dienophiles *trans*-cyclooctene (TCO) and *endo*-bicyclo[6.1.0]non-4-yn-9-ylmethanol (BCN). Fox and co-workers reported that TCO^11^ reacts 440 times faster than BCN^12^ with diene **1**. In accordance with FMO theory, the higher HOMO energy of TCO makes it a more reactive dienophile in inverse electron-demand Diels–Alder reactions compared to BCN.^13,14^ By contrast, TCO reacts 110 times slower than BCN when reacted with **3**, as recently reported by van Delft and co-workers.^15^ This unexpected reactivity difference prompted us to investigate the reactivities of TCO and BCN with a third bioorthogonal diene, **2**_**Ethyl**_. Stopped-flow kinetic experiments (see Supporting Information) show that BCN is only twice as reactive toward **2**_**Ethyl**_ as TCO. To rationalize these reactivity trends, we have analyzed interactions of the frontier and subjacent molecular orbitals and discovered that secondary orbital interactions promote the reactivity of BCN toward **2**_**Ethyl**_ and even more so toward **3**.

**Scheme 1:**
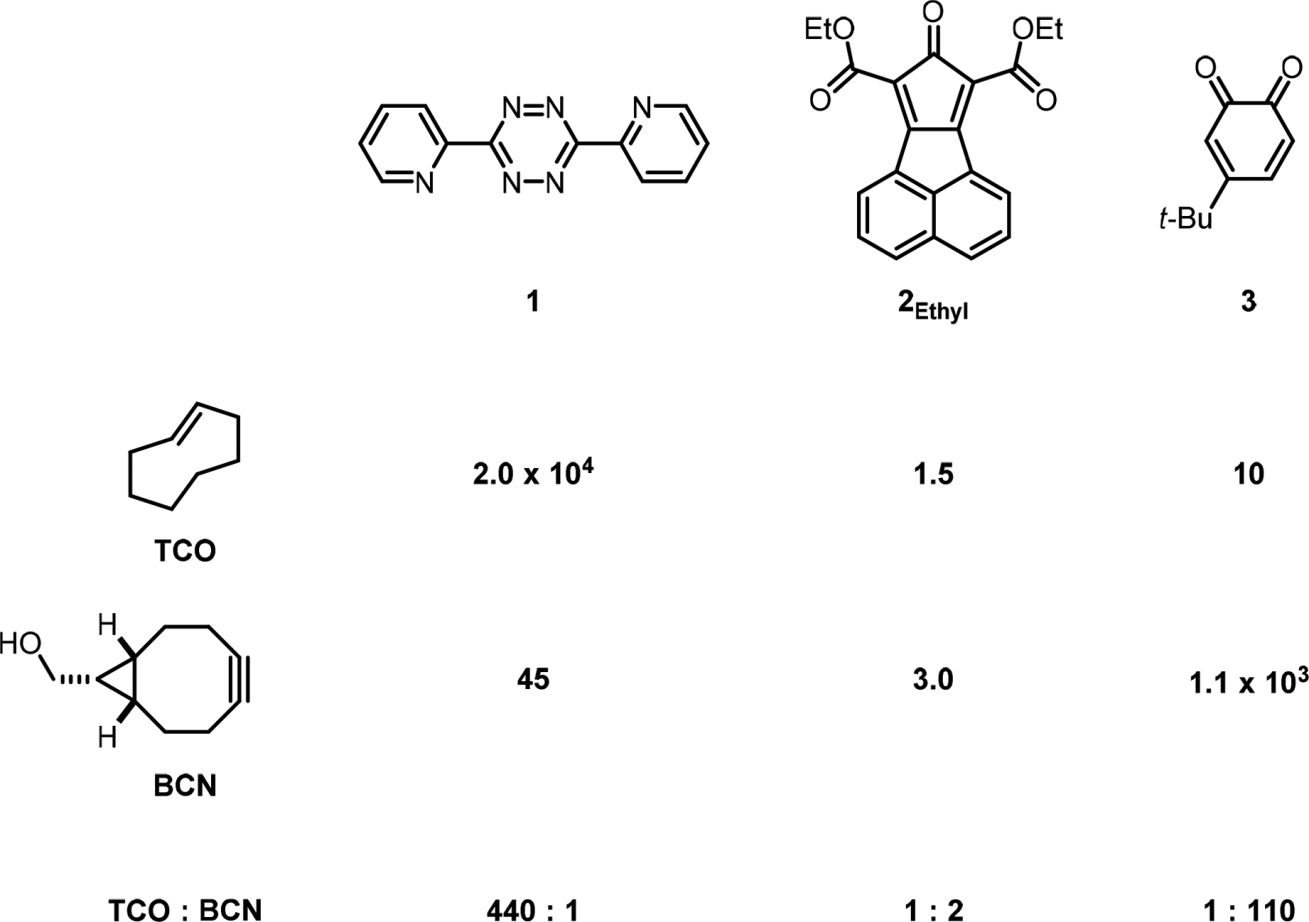
Second-Order Rate Constants (M^–1^ s^–1^) for the Diels–Alder Reactions of **1**,^11,12^**2**_**Ethyl**_, and **3**(15) with TCO and BCN, and the Relative Rates of TCO and BCN with Each Diene

Computational investigations were performed using TCO, a truncated BCN (BCN*), dipyridyl tetrazine **1**, the dimethyl ester **2**_**Methyl**_, and the *ortho*-benzoquinone **3** (Scheme2). The M06-2X^16^ functional with the 6-31G(d) basis set was used for geometry optimizations. Energies were calculated using the larger 6-311++G(d,p) basis set. The transition state structures and the calculated Gibbs activation free energies (Δ*G*^‡^) for the Diels–Alder reactions of **1**, **2**_**Methyl**_, and **3** with TCO and BCN* are shown in Figure 1. The activation free energies of these bioorthogonal reactions range from 12 to 18 kcal/mol. In agreement with experimental results, the computed rate constants predict that **1** will react 440 times faster with TCO than BCN*, that **2**_**Methyl**_ has similar reactivity toward TCO and BCN*, and that **3** will react with BCN* 440 times faster than with TCO. These results are in reasonable agreement with the experimental results described earlier. Calculations using the implicit solvent model SMD show the same trends as obtained in gas phase and are provided in the Supporting Information.

**Scheme 2:**
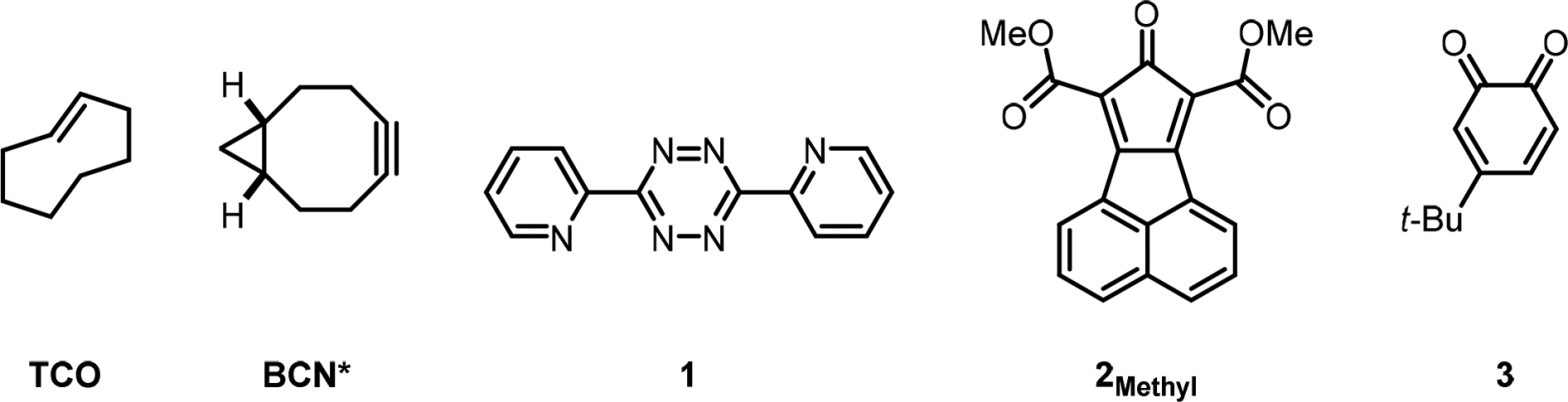
Structures Used in the Computational Study

**Figure 1:**
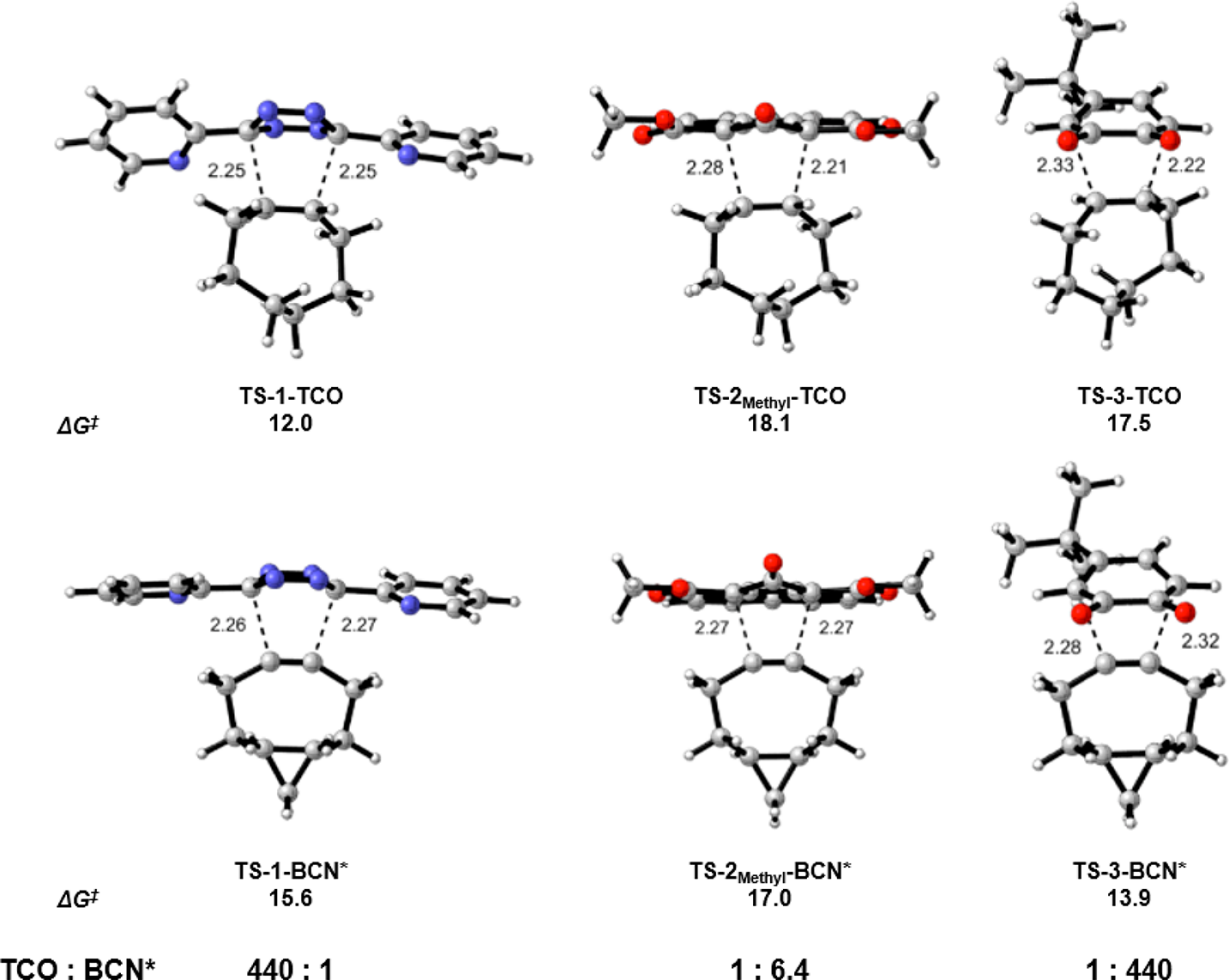
Transition state structures, Gibbs activation free energies, and predicted relative reaction rates for the Diels–Alder reactions of **1**, **2**_**Methyl**_, and **3** with TCO and BCN*. Bond lengths are reported in Å and energies are reported in kcal/mol.

**1**, **2**_**Methyl**_, and **3** are highly electron-deficient dienes that react with the electron-rich dienophiles TCO and BCN* through an inverse electron-demand DA mechanism. The primary orbital interactions involve the HOMO of TCO or BCN* interacting with the LUMO of **1**, **2**_**Methyl**_ or **3**. The HOMOs of TCO and BCN* and the LUMOs of 1–3 are shown in Figure 2. The HOMO energies of TCO and BCN* are −9.0 and −9.6 eV, respectively. With a higher lying HOMO, the strength of the primary FMO interactions with TCO are more favorable than with BCN*, and the primary FMO interactions predict that TCO should be more reactive than BCN* in inverse electron-demand Diels–Alder reactions.

**Figure 2:**
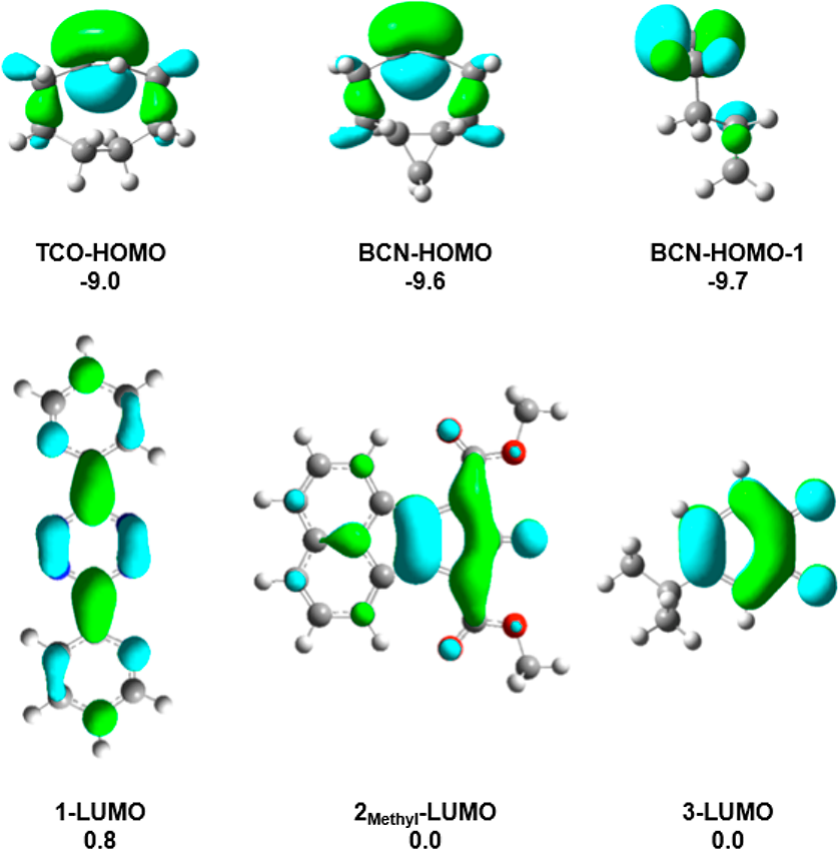
HOMOs of TCO and BCN*, HOMO-1 of BCN*, and LUMOs of **1**, **2**_**Methyl**_, and **3** generated with isovalues of 0.04. Molecular orbital energies are provided in electron volts (eV).

To understand the origin of the differences in the Diels–Alder reactivities of TCO and BCN* toward **1**, **2**_**Methyl**_, and **3**, we performed a distortion/interaction analysis.^3^ Within this analysis the energy of the system along the reaction coordinate gets dissected into two contributing factors. The distortion energy Δ*E*_dist_ is the energy required to geometrically deform the ground state geometries of the reactants. The interaction energy Δ*E*_int_ represents the energy of the interactions that occur between the distorted reactants. These include the orbital, electrostatic, and steric interactions. The distortion/interaction analysis was performed along the IRC defined by the distance of the shortest forming carbon–carbon bond from a forming bond length of 2.6 Å up to the transition state geometry.

The results of the distortion/interaction analysis are shown in Figure 3. For the Diels–Alder reactions of TCO and BCN* with **1**, both the distortion and interaction energies are more favorable for the reaction with TCO. For reactions with diene **2**_**Methyl**_, the distortion energies favor the reaction with TCO, but are offset by the interaction energies, which are more stabilizing with BCN*. This results in similar reactivities of TCO and BCN* toward **2**_**Methyl**_. For the cycloaddition of TCO and BCN* with **3**, the distortion energies along the IRC are nearly identical and the higher reactivity of BCN* toward **3** can be attributed to the more favorable interaction energies.

**Figure 3:**
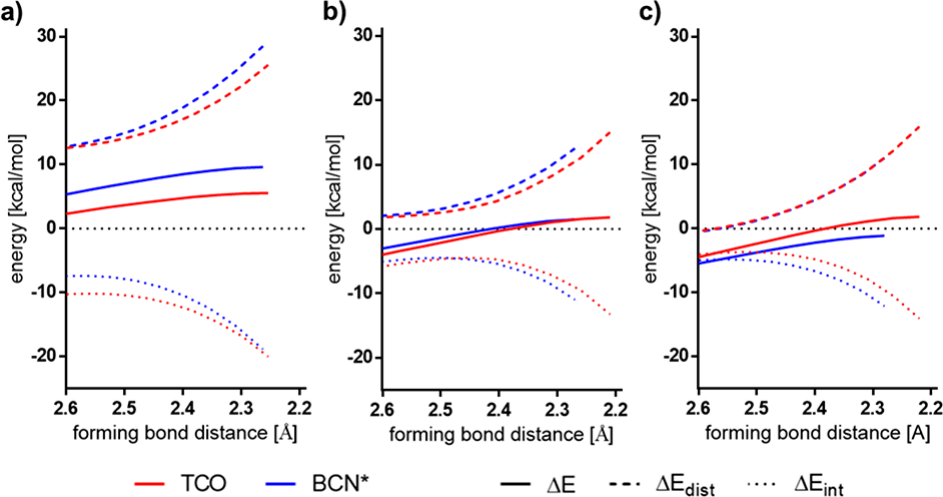
Distortion/interaction analysis for the Diels–Alder reactions of TCO (red) and BCN* (blue) with (a) **1**, (b) **2**_**Methyl**_, and (c) **3**.

Secondary orbital interactions are known to influence the reactivity and stereoselectivity of Diels–Alder reactions.^17−21^ The BCN* HOMO-1 is the nonreacting, out-of-plane π-bond and is nearly degenerate to the HOMO (Figure 2). Secondary orbital interactions involving overlap of the HOMO-1 of BCN* with the LUMOs of **1**, **2**_**Methyl**_, and **3** are illustrated in Figure 4 with a schematic orbital diagram. The **2**_**Methyl**_-BCN* transition state is stabilized by secondary orbital interactions associated with the orbital overlap of the *endo* facing lobe of the HOMO-1 in BCN* with the LUMO of **2**_**Methyl**_ at the C_3_ and C_4_ carbons, and between the *exo* facing lobe of the BCN* HOMO-1 with the C_1_ carbonyl carbon in the LUMO of **2**_**Methyl**_. These secondary orbital interactions are also present in the transition state 3-BCN*, in addition to an interaction involving the overlap of the *exo* facing lobe of the BCN* HOMO-1 with the additional carbonyl carbon in the LUMO of **3**. Although the HOMO-1 of BCN* is not a frontier molecular orbital, overlap of the BCN* HOMO-1 with the LUMOs of **2**_**Methyl**_ and **3** at the transition state is significantly stabilizing and has an important effect on the Diels–Alder reactivities.

**Figure 4:**
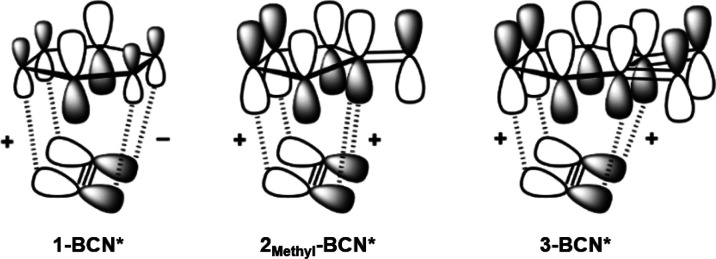
Schematic illustration of the constructive (+) and destructive (−) secondary orbital interactions between the HOMO-1 of BCN* and the LUMOs of **1**, **2**_**Methyl**_, and **3**.

The LUMO density at the nitrogen atoms in **1** is significantly smaller compared to the carbon atoms in the LUMOs of **2**_**Methyl**_ and **3**, and the in-phase interaction of the BCN* HOMO-1 with the LUMO density across the N–N bond in **1** is counteracted by an out-of-phase interaction with the LUMO density across the opposite N–N bond. Because of the mismatched symmetry of the BCN* HOMO-1 and the LUMO of **1**, the secondary orbital interactions result in no stabilization, and the relative strengths of the primary orbital interactions dictate reactivity, resulting in a less reactivate BCN compared to TCO in tetrazine cycloadditions.

We have studied the inverse electron-demand Diels–Alder reactions of BCN and TCO toward **1**, **2**_**Methyl**_, and **3** and rationalize why BCN, despite having a lower HOMO energy compared to TCO, shows similar reactivity toward **2**_**Methyl**_, and is even more reactive than TCO toward **3**. Secondary orbital interactions between the HOMO-1 of alkynes and the LUMOs of dienes like **2**_**Methyl**_ and **3** significantly stabilize the transition state and promote reactivity. The stabilization from the secondary orbital interactions in the DA reactions of **2**_**Methyl**_ with BCN results in the similar reactivities of BCN and TCO. The additional carbonyl group in **3** further strengths the secondary orbital interactions between the HOMO-1 of BCN and the LUMO of **3**. This additional stabilization results in **3** being more reactive toward BCN than TCO. Diels–Alder reactions of alkynes play an important role in bioorthogonal chemistry, and secondary orbital interactions of the alkyne HOMO-1 should be considered in the development of new bioorthogonal reactions.

## Supporting Information Available

Experimental details, Cartesian coordinates, and energies of all optimized structures and transition structures (PDF)
