# Correction to “Secondary Orbital Interactions Enhance the Reactivity of Alkynes in Diels–Alder Cycloadditions”

**DOI:** 10.1021/jacs.9b11320

**Published:** 2019-11-06

**Authors:** Brian J. Levandowski, Dennis Svatunek, Barbara Sohr, Hannes Mikula, K. N. Houk

Page 2224. The experimental rate constant from ref 11 for the Diels–Alder reaction of **1** with TCO in methanol at room temperature was given incorrectly in Scheme 1. The correct value is 1.1 × 10^3^ M^–1^ s^–1^. The corrected Scheme 1 is provided here.

**Scheme 1:**
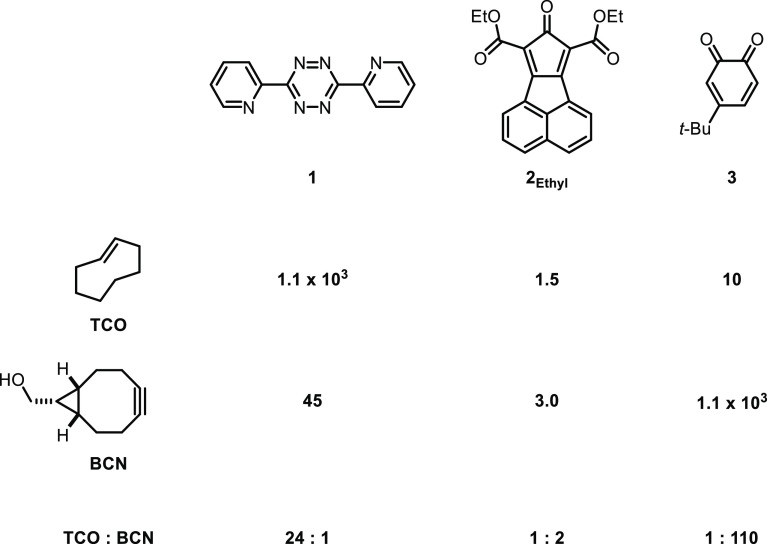
Second-Order Rate Constants (M^–1^ s^–1^) for the Diels–Alder Reactions of **1**, **2**_Ethyl_, and **3** with TCO and BCN, and the Relative Rates of TCO and BCN with Each Diene

In addition, on page 2224 the reactivity difference of TCO and BCN toward diene **1** was incorrectly described as “440 times faster”; it should read “24 times faster”.

