# Supplementary material for: Secondary Orbital Interactions Enhance the Reactivity of Alkynes in Diels–Alder Cycloadditions
Source: J Am Chem Soc. 2019 Jan 29;141(6):2224–7. doi: 10.1021/jacs.8b13088 (PMC8830044; doi:10.1021/jacs.8b13088)
Supplement: Supplementary file 1 — ja8b13088_si_001.pdf [file ja8b13088_si_001.pdf]

Secondary Orbital Interactions Enhance the Reactivity of Alkynes in Diels-Alder Cycloadditions

Brian J. Levandowski,[†,⊥] Dennis Svatunek,[†,‡,⊥] Barbara Sohr,[‡] Hannes Mikula,[‡] and K. N. Houk[†]

[†]Department of Chemistry and Biochemistry, University of California, Los Angeles, California 90095

[‡]Institute of Applied Synthetic Chemistry, TU Wien, Vienna, Austria

Table of Contents

Experimental Details……………………………………………………………...…..S1

Table S1. Experimental concentrations and second order rate constants………...….S1

Computational Details…………………………………………………………….…..S1

Table S2. M06-2X/6-31G(d) optimized Cartesian coordinates…………………...…..S2

Table S3. Energetics ……………………………………………………………………S11

Table S3. Energetics in solvents…..……………………………………………………S11

References……………….……………………………...………………………………S12

Experimental Details

Unless otherwise noted, all reagents were purchased from commercial suppliers and used without further purification. Diethyl 8-oxo-8H-cyclopent[a]acenaphthylene-7,9-dicarboxylate (**2_{Ethyl}**),[1] *trans*-cyclooctene (TCO),[2] pegylated TCO **6**,[3] and BCN[4] were prepared following known procedures. 1,4-Dioxane was dried using PURESOLV-columns (Innovative Technology Inc.). All other solvents were distilled prior to use. Drying of organic solvents after extraction was performed using anhydrous Na_2SO_4 and subsequent filtration. Reactions were carried out under an atmosphere of argon in air-dried glassware with magnetic stirring. Sensitive liquids were transferred via syringe. Thin layer chromatography was performed using TLC alumina plates (Merck, silica gel 60, fluorescence indicator F254, or Merck, aluminium oxide neutral, fluorescence indicator F254). Normal phase flash column chromatography was performed using a Büchi Sepacore Flash System (2 x Büchi Pump Module C-605, Büchi Pump Manager C-615, Büchi UV Photometer C-635, Büchi Fraction Collector C-660) or on a Grace Reveleris X2 Prep using silica gel 60 (40-63 µm) as obtained from Merck and distilled or re-distilled solvents. ^1H and ^{13}C NMR spectra were recorded on a Bruker AC 200, Bruker Avance UltraShield 400 spectrometer at 20 °C or Bruker Bruker Avance III HD 600.

For kinetic investigations the reactions between **2_{Ethyl}** and TCO (Scheme S1a) and **2_{Ethyl}** and BCN (Scheme S1b) were used. The product of **2_{Ethyl}** and BCN is known to eliminate CO spontaneously to yield structure **5**.[5] To determine the reaction product of derivatives of **2** and trans-cyclooctenes the reaction of **2_{Ethyl}** and TCO was performed at a preparative scale. In addition, an LC-MS study was conducted on the reaction between **2_{Ethyl}** and pegylated TCO to confirm the reaction product (Scheme S2).

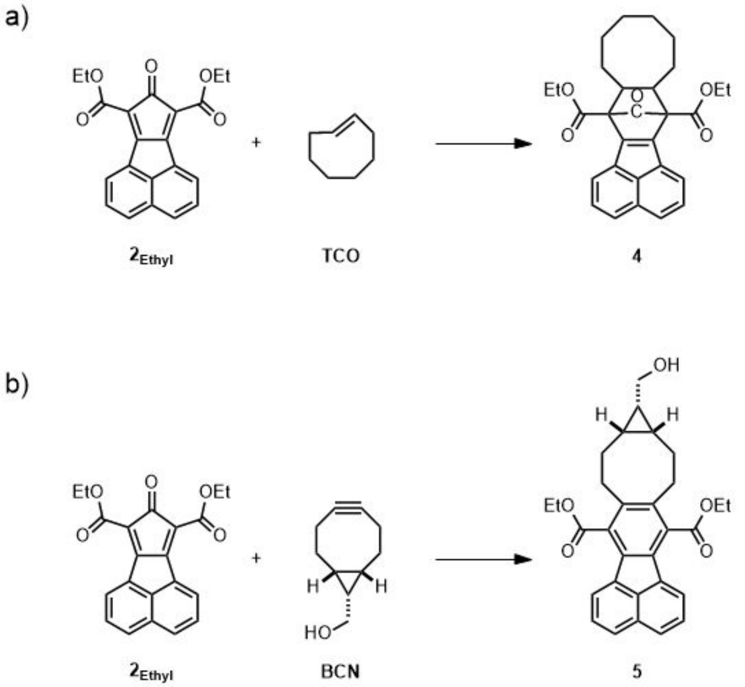

Scheme S1. a) reaction between **2_{Ethyl}** and TCO; b) reaction between **2_{Ethyl}** and BCN.

Synthesis of 4

$\mathbf{2_{Ethyl}}$ (320 mg, 0.92 mmol) was dissolved in 20 mL dry 1,4-dioxane and TCO (110 mg, 1 mmol) was added. A color change from dark red to yellow was observed. Full conversion was confirmed by TLC. The solvent was evaporated and the crude was purified by column chromatography (90 g silica gel, 5 - 20% ethyl acetate in hexanes) to yield 95 mg of a yellow oil. ^{1}H NMR (600 MHz, THF-d8) δ 8.04 (d, J = 6.8 Hz, 1H), 7.88 (d, J = 6.8 Hz, 1H), 7.86 - 7.83 (m, 2H), 7.56 (dd, J = 7.1, 4.4 Hz, 1H), 7.54 (dd, J = 6.6, 4.6 Hz, 1H), 4.43 - 4.46 (m, 4H), 2.97 - 2.91 (m, 1H), 2.06 - 1.98 (m, 2H), 1.90 - 1.81 (m, 1H), 1.80 - 1.75 (m, 1H), 1.64 - 1.48 (m, 6H), 1.39 - 1.28 (m, 9H); ^{13}C NMR (150 MHz, THF-d8) δ 188.3, 168.1, 168.0, 141.7, 139.1, 135.8, 133.6, 129.9, 129.7, 128.93, 128.86. 128.35, 128.34, 126.1, 125.6, 67.8, 65.1, 61.9, 61.6, 49.1, 46.1, 32.2, 31.9, 27.8, 27.4, 24.4, 23.4, 14.7, 14.6;

The ^{13}C signal at 188.3 ppm corresponds to the carbonyl group still present in the product. To confirm that the cycloaddition product of $\mathbf{2_{Ethyl}}$ and TCOs does not undergo cyclo-elimination of CO an additional reaction between $\mathbf{2_{Ethyl}}$ and pegylated TCO **6** was conducted to confirm the presence of the CO group by MS.

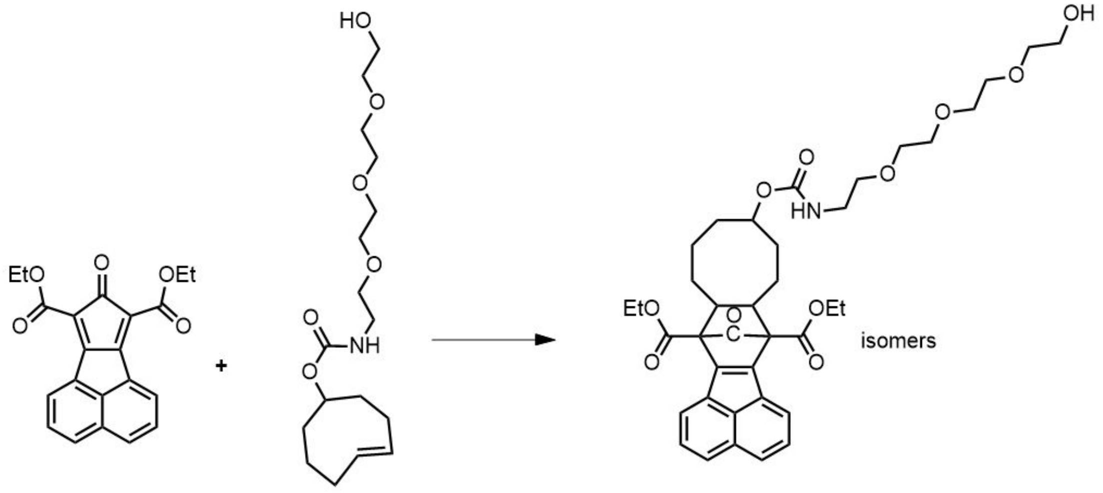

$\mathbf{2_{Ethyl}}$ **6** **7**

Scheme S2. Synthesis of **7**

$\mathbf{2_{Ethyl}}$ (105 mg, 0.3 mmol) was dissolved in dry 1,4-dioxane (7 mL) and **6** was added as solution in 1,4-dioxane (154 mg, 0.45 mmol, in 1.5 mL). A color change from red to yellow was observed. LC-MS revealed full conversion to a single product. The observed $[M+H]^+$ of 694.3 corresponds to structure **7** without elimination of CO.

Kinetic measurements

Kinetic measurements were performed using a temperature controlled SX20 stopped-flow spectrophotometer (Applied Photophysics, UK) equipped with LED light sources. All measurements were performed in triplicates using pseudo first order conditions with an excess of dienophile in dry 1,4-dioxane at 25±0.1°C. Data analysis was performed in PRISM 6 (GraphPad). Measurements of the reaction between $\mathbf{2}_{\mathbf{Ethyl}}$ and TCO were performed in absorbance mode using a 535 nm LED and following the decrease in absorbance. Measurements of the reaction between $\mathbf{2}_{\mathbf{Ethyl}}$ and BCN were performed in fluorescence mode using an excitation wavelength of 365 nm in combination with a 400 nm long pass filter, following the increase in fluorescence >400 nm. Table S1 shows the used concentrations, and observed and second order rate constants.

Table S1. Concentrations used in kinetic experiment, observed and second order rate constants.

Diene	Dienophile	Concentration dienophile [mM]	Concentration diene [mM]	Observed rate constant [s^{-1}]	Second order rate constant [$M^{-1}s^{-1}$]
$\mathbf{2}_{\mathbf{Ethyl}}$	TCO	1.9	0.058	0.00296 ± 0.000046	1.50 ± 0.024
$\mathbf{2}_{\mathbf{Ethyl}}$	BCN	2.0	0.058	0.00607 ± 0.000005	3.04 ± 0.0025

Computational Details

Calculations were performed using Gaussian09 Rev.D01. M06-2X/6-31G(d) was used for geometry optimization of minima and transition states followed by frequency analysis at the same level of theory, confirming the presence of a minima with no imaginary frequency or a transition state with exactly one imaginary frequency. Single point energy calculations were performed at the M06-2X/6-311++G(d,p) level of theory using M06-2X/6-31G(d) optimized geometries. Gibbs free energies were calculated by applying thermal corrections of the M06-2X/6-31G(d) frequency analysis to M06-2X/6-311++G(d,p)//M06-2X/6-31G(d) electronic energies.

Table S2. M06-2X/6-31G(d) optimized Cartesian coordinates

BCN*

C	-0.60401000	1.76198400	-0.15212800
C	0.60388100	1.76202500	-0.15209800
C	-1.92946900	1.14836100	-0.03269500
C	-1.66243500	-0.29025200	0.47889500
H	-2.61684200	-0.82146600	0.58824300
H	-1.21973200	-0.21991900	1.47921300
C	-0.75804300	-1.09575400	-0.43626100
C	0.75812000	-1.09570200	-0.43626700
C	1.92938600	1.14849600	-0.03269500
H	2.58245900	1.68594300	0.66274300
H	2.44121900	1.12057900	-1.00208800
H	-2.44132400	1.12040300	-1.00207500
H	-2.58256200	1.68576900	0.66275500
C	1.66246000	-0.29014000	0.47888900
H	2.61690600	-0.82128500	0.58822900
H	1.21975800	-0.21984300	1.47921000
H	-1.17998500	-1.21819300	-1.43326600
H	1.18006700	-1.21810900	-1.43327300
C	0.00008100	-2.29030200	0.07629700
H	0.00011000	-3.21222400	-0.49582300
H	0.00009100	-2.43395100	1.15451500

TCO

C	0.53224700	-1.35823000	-0.40081400
C	-0.53225200	-1.35823100	0.40081300
C	1.87012300	-0.90135500	0.08160500
C	1.88190600	0.63564100	-0.13550300
H	2.79098100	1.04825600	0.31889000
H	1.95634100	0.84157000	-1.21172200
C	0.65032200	1.37612400	0.43050100
C	-0.65031700	1.37612400	-0.43050200

C	-1.87012700	-0.90135000	-0.08160400
H	-2.71606600	-1.35793700	0.44339800
H	-1.98062100	-1.11343800	-1.15202200
H	2.71606000	-1.35794500	-0.44339800
H	1.98061600	-1.11344500	1.15202200
C	-1.88190300	0.63564600	0.13550500
H	-2.79097700	1.04826500	-0.31888700
H	-1.95633500	0.84157500	1.21172400
H	0.95017200	2.41884800	0.58536500
H	-0.42951600	0.98415200	-1.43205800
H	0.42952000	0.98415700	1.43205800
H	-0.95016500	2.41884800	-0.58537100
H	-0.37373800	-1.33456800	1.48125600
H	0.37373300	-1.33456300	-1.48125700

3

C	1.451326000	1.638861000	-0.001926000
C	0.282565000	-0.995233000	-0.008285000
H	1.888272000	2.632556000	0.001260000
H	-0.124290000	-2.002770000	-0.006369000
C	-0.504606000	0.100832000	-0.011892000
C	0.123203000	1.439295000	-0.005971000
C	2.386274000	0.498238000	-0.002267000
C	1.752338000	-0.912993000	-0.002296000
O	2.466223000	-1.890949000	0.002634000
O	3.589016000	0.625387000	-0.001981000
H	-0.530201000	2.304594000	-0.006703000
C	-2.032637000	-0.043478000	-0.001334000
C	-2.469836000	-0.927062000	-1.182006000
H	-2.030283000	-1.926568000	-1.123831000
H	-3.559383000	-1.037768000	-1.178470000
H	-2.172615000	-0.480339000	-2.136265000
C	-2.453455000	-0.709739000	1.320222000
H	-2.146932000	-0.104456000	2.179568000
H	-3.542662000	-0.822808000	1.351561000
H	-2.004339000	-1.701414000	1.426925000
C	-2.758415000	1.302543000	-0.116851000
H	-2.546868000	1.957056000	0.735228000
H	-2.494843000	1.832090000	-1.038843000
H	-3.838308000	1.126735000	-0.133650000

TS-3-BCN*

C	-0.909047000	1.701356000	-1.306021000
C	-1.505202000	0.312338000	1.010075000
C	0.972619000	0.564622000	-0.550751000
C	0.675758000	-0.074010000	0.471163000

C	1.984015000	1.050484000	-1.508384000
C	3.378570000	0.571758000	-1.062222000
H	3.616826000	1.038087000	-0.099704000
H	4.124827000	0.932803000	-1.780835000
C	3.477690000	-0.935201000	-0.955831000
C	3.115830000	-1.727211000	0.281731000
C	2.616801000	-1.112504000	1.572215000
H	3.089638000	-0.137904000	1.736678000
H	2.913521000	-1.751417000	2.413329000
C	1.088660000	-0.925302000	1.604877000
H	0.785062000	-0.455173000	2.548866000
H	0.578476000	-1.896410000	1.552057000
H	1.755979000	0.684317000	-2.517326000
H	1.951355000	2.147051000	-1.548410000
C	4.555187000	-1.579205000	-0.128253000
H	3.243635000	-1.446011000	-1.888943000
H	2.669938000	-2.698262000	0.069912000
H	5.085720000	-2.438670000	-0.523841000
H	5.167053000	-0.914578000	0.477301000
H	-0.603531000	2.196255000	-2.224978000
H	-1.650048000	-0.245915000	1.930662000
C	-2.153613000	-0.045613000	-0.156079000
C	-1.843088000	0.692501000	-1.324196000
C	-0.592797000	2.436494000	-0.060734000
C	-0.956234000	1.674879000	1.224745000
O	-0.778155000	2.149546000	2.323267000
O	-0.084390000	3.534793000	-0.037708000
H	-2.220746000	0.349627000	-2.283899000
C	-2.989375000	-1.319424000	-0.278991000
C	-3.222966000	-1.986993000	1.079331000
H	-3.860097000	-2.867540000	0.948786000
H	-2.283147000	-2.322994000	1.531188000
H	-3.720735000	-1.309228000	1.780812000
C	-2.246937000	-2.315334000	-1.187862000
H	-2.807338000	-3.254375000	-1.257691000
H	-2.121072000	-1.921659000	-2.201079000
H	-1.251544000	-2.534156000	-0.785590000
C	-4.354555000	-0.974893000	-0.894285000
H	-4.952283000	-1.885684000	-1.011131000
H	-4.907295000	-0.281407000	-0.252338000
H	-4.251729000	-0.513920000	-1.881449000

TS-3-TCO

C	3.823146000	-0.674018000	0.687131000
C	3.238530000	-1.959624000	0.035623000

C	2.741136000	-1.834692000	-1.416379000
C	1.325964000	-1.245460000	-1.594980000
C	1.224237000	0.050934000	-0.839224000
C	0.965874000	0.009533000	0.530379000
C	1.824153000	0.788756000	1.484775000
C	3.039952000	-0.079679000	1.871579000
H	0.579621000	-1.948488000	-1.201052000
H	1.123227000	-1.119592000	-2.664586000
H	2.755594000	-2.824453000	-1.888489000
H	3.453553000	-1.217711000	-1.979976000
H	2.434272000	-2.365864000	0.663516000
H	4.021736000	-2.725498000	0.039392000
H	4.829898000	-0.906712000	1.050269000
H	3.962621000	0.100861000	-0.078634000
H	2.702882000	-0.898950000	2.520720000
H	3.713867000	0.535010000	2.479300000
H	2.165214000	1.713981000	1.003789000
H	1.276981000	1.085307000	2.386892000
H	0.573695000	-0.927875000	0.923129000
H	1.889350000	0.846129000	-1.180918000
C	-1.200076000	0.794079000	0.853008000
H	-1.356586000	0.691582000	1.922944000
C	-1.907364000	0.017675000	-0.058745000
C	-1.568836000	0.144600000	-1.419560000
H	-1.993277000	-0.545668000	-2.144110000
C	-0.537371000	0.980089000	-1.820614000
H	-0.232367000	1.021088000	-2.863603000
C	-0.220910000	2.189795000	-1.027698000
C	-0.577295000	2.069824000	0.463829000
O	0.309540000	3.178667000	-1.477179000
O	-0.315261000	2.958892000	1.245126000
C	-2.876999000	-1.085018000	0.373999000
C	-3.137542000	-1.063500000	1.883021000
H	-3.870681000	-1.835419000	2.138212000
H	-2.226429000	-1.271340000	2.454542000
H	-3.536154000	-0.097049000	2.208348000
C	-2.291976000	-2.459261000	0.003083000
H	-2.974785000	-3.257779000	0.314601000
H	-2.133267000	-2.554535000	-1.075900000
H	-1.330080000	-2.622248000	0.503134000
C	-4.216002000	-0.886676000	-0.354332000
H	-4.921227000	-1.673254000	-0.063902000
H	-4.656713000	0.082335000	-0.099281000
H	-4.096898000	-0.928475000	-1.441287000

1

C	-1.28011900	-0.01987300	0.00000100
C	1.28014600	0.01986000	-0.00000600
C	-2.76851500	-0.01001400	0.00000000
C	-3.45530000	1.20683300	0.00000000
C	-4.84470900	1.17598700	0.00000100
H	-2.90193900	2.13800500	0.00000000
C	-4.70517900	-1.20976900	0.00000200
C	-5.48829100	-0.05541100	0.00000200
H	-5.41327500	2.10067600	0.00000100
H	-5.17231100	-2.19211900	0.00000300
H	-6.57050200	-0.12836100	0.00000200
C	2.76850300	0.01004500	-0.00000100
C	3.45521000	-1.20677400	-0.00000100
C	4.70517300	1.20974400	0.00000300
C	4.84466700	-1.17601600	0.00000200
H	2.90183100	-2.13794400	-0.00000300
C	5.48828000	0.05531300	0.00000400
H	5.17242300	2.19204500	0.00000400
H	5.41312500	-2.10076900	0.00000200
H	6.57048900	0.12827600	0.00000600
N	0.64220900	1.19917300	-0.00000300
N	-0.66330200	1.17048500	-0.00000100
N	-0.64210700	-1.19913900	-0.00000100
N	0.66328500	-1.17048400	-0.00000300
N	-3.37593500	-1.19949500	0.00000100
N	3.37598800	1.19955200	0.00000000

TS-1-BCN*

C	1.22713500	-1.41648300	0.07347400
N	0.61979300	-1.57138400	1.29096400
N	-0.65856700	-1.56018400	1.30545000
C	-1.26402300	-1.39817000	0.08560300
N	-0.66098900	-1.92055500	-1.02764400
N	0.61707200	-1.92992900	-1.04244500
C	5.43680100	-1.26397100	0.01331000
C	4.80207800	-1.34049400	1.24802900
C	3.41403800	-1.39393000	1.28861200
C	2.71554800	-1.36586800	0.07855200
N	3.31410900	-1.27904100	-1.11264800
C	4.64440600	-1.23623800	-1.13270100
H	6.51776500	-1.22224600	-0.06657600
H	5.37850200	-1.36232100	2.16786400
H	2.86647100	-1.46466400	2.22086100
H	5.10201700	-1.16851100	-2.11727900
C	-5.47030200	-1.16692600	0.07757100

C	-4.67759100	-0.81030100	1.16674100
N	-3.34828900	-0.88089600	1.16843200
C	-2.75147800	-1.33123300	0.06133700
C	-3.45031700	-1.71067400	-1.08792300
C	-4.83705200	-1.62377300	-1.07304100
H	-6.55031200	-1.08377200	0.13508400
H	-5.13379500	-0.44284500	2.08340600
H	-2.90373300	-2.06706300	-1.95288300
H	-5.41351100	-1.91168900	-1.94685800
C	0.60913000	0.73533000	-0.27904700
C	-0.62497200	0.75216700	-0.21263700
C	1.88807600	1.44976500	-0.46778900
C	1.60007200	2.94162400	-0.71526700
H	1.03849500	3.04372700	-1.65073800
H	2.55632600	3.45797100	-0.86658400
C	0.85340000	3.60182600	0.42306300
C	-0.65236000	3.62617100	0.53930900
C	-1.58744700	2.99978500	-0.47214000
H	-1.17282400	3.09087600	-1.48247400
H	-2.53603500	3.55126200	-0.47209600
C	-1.88851700	1.51787100	-0.18423700
H	-2.60093700	1.12614400	-0.91921700
H	-2.35817800	1.40099800	0.79932100
H	2.52246300	1.32700100	0.41978300
H	2.43813800	1.01757200	-1.30969600
C	0.10001100	4.88492100	0.20533200
H	1.37022400	3.52153100	1.37826500
H	-1.02044700	3.55866800	1.56176900
H	0.17100800	5.67354700	0.94670600
H	0.02664500	5.23934400	-0.82032700

TS-1-TCO

C	0.23362300	3.70623400	0.74244900
C	-0.23403500	3.70636000	-0.74222200
C	-1.54414800	2.96083700	-1.05909800
C	-1.41341000	1.42808800	-1.21305000
C	-0.68745800	0.91040800	-0.00607800
C	0.68746900	0.91042400	0.00586400
C	1.41342000	1.42809500	1.21284300
C	1.54389100	2.96089300	1.05909800
C	1.24213100	-1.27384500	-0.05417200
N	0.69009800	-1.61507700	1.14793000
C	-1.24202600	-1.27383500	0.05428300
N	-0.59052700	-1.60558600	1.21268200
H	-0.83724800	1.19207600	-2.11602000
H	-2.41186000	0.99218000	-1.32919900

H	-1.96956100	3.35856900	-1.98801200
H	-2.27581100	3.17485500	-0.26882800
H	0.56648100	3.31784000	-1.38589300
H	-0.37485700	4.74777700	-1.05162600
H	0.37422000	4.74760200	1.05211600
H	-0.56680400	3.31738000	1.38602500
H	2.27542500	3.17513200	0.26876900
H	1.96935800	3.35856800	1.98801100
H	0.83738400	1.19185200	2.11583200
H	2.41195800	0.99233500	1.32882000
H	1.20626200	1.01693500	-0.94839000
H	-1.20619400	1.01697100	0.94820100
C	5.42211200	-0.86409600	-0.30582800
C	4.89495700	-1.47687800	0.82530700
C	3.51693200	-1.62519200	0.92855400
C	2.72266200	-1.15061600	-0.11720000
N	3.21605200	-0.54967700	-1.20360400
C	4.53828900	-0.41844300	-1.28672500
H	6.49098700	-0.72678200	-0.42930500
H	5.54644100	-1.83685200	1.61574100
H	3.04808400	-2.09328400	1.78606500
H	4.91153300	0.07363000	-2.18222000
C	-5.42205200	-0.86429500	0.30572600
C	-4.53830400	-0.41865200	1.28669500
N	-3.21605700	-0.54983400	1.20364800
C	-2.72257700	-1.15070200	0.11724900
C	-3.51676700	-1.62525400	-0.92858000
C	-4.89480300	-1.47700500	-0.82540500
H	-6.49093900	-0.72703100	0.42914600
H	-4.91162200	0.07337200	2.18218600
H	-3.04785600	-2.09329200	-1.78608500
H	-5.54622400	-1.83696700	-1.61589700
N	-0.68999200	-1.61522000	-1.14778300
N	0.59062500	-1.60574900	-1.21254300

2_{Methyl}

C	2.08267700	2.41506500	-0.00038600
C	3.50173600	2.42971500	-0.00063400
C	4.25494700	1.27417000	-0.00076200
C	3.62415000	0.00000000	-0.00064800
C	2.22387700	0.00000000	-0.00040400
C	1.44609300	1.19396700	-0.00027100
C	4.25494700	-1.27417000	-0.00075400
C	3.50173600	-2.42971500	-0.00062000
C	2.08267700	-2.41506500	-0.00037200
C	1.44609300	-1.19396700	-0.00026400

C	0.05549600	0.75489800	-0.00002400
C	0.05549600	-0.75489800	-0.00001900
C	-1.20874000	-1.21620100	0.00021100
C	-2.12542200	0.00000000	0.00037100
C	-1.20874000	1.21620100	0.00020300
C	-1.59124100	2.63749200	0.00027100
C	-1.59124100	-2.63749200	0.00028800
O	-2.91342900	-2.81804300	0.00055700
O	-0.78987500	-3.54868000	0.00012900
O	-3.32667000	0.00000000	0.00058400
O	-0.78987400	3.54868000	0.00010500
O	-2.91342900	2.81804300	0.00053700
C	-3.33331100	4.18102700	0.00061400
C	-3.33331100	-4.18102700	0.00064600
H	1.51044500	3.33498300	-0.00028800
H	4.00845800	3.38950400	-0.00072500
H	5.34012100	1.33184900	-0.00095100
H	5.34012100	-1.33184900	-0.00094400
H	4.00845800	-3.38950400	-0.00070600
H	1.51044500	-3.33498300	-0.00026900
H	-4.42124800	4.15166400	0.00084100
H	-2.95902900	4.69416100	0.88920800
H	-2.95940000	4.69414800	-0.88814500
H	-4.42124800	-4.15166400	0.00087500
H	-2.95940200	-4.69415500	-0.88810900
H	-2.95902700	-4.69415400	0.88924400

TS-2$_{\mathbf{Methyl}}$-TCO

C	-0.27134700	-1.06336300	-1.17351100
C	-0.41488800	1.29947200	-0.93234500
C	-1.57061700	0.50560200	0.86077900
C	-1.49230400	-0.87584800	0.65955100
C	-0.72414300	-1.73026800	1.62591100
C	-2.90956600	1.18298400	0.82911500
C	-1.64032900	-2.10416000	2.81003000
C	-3.59308200	1.02636100	2.20747900
C	-3.64748700	-0.40358300	2.77203500
C	-2.36052600	-0.91882300	3.47651800
C	0.87182100	0.87032100	-0.65177200
C	0.96401200	-0.55220300	-0.80898700
C	-1.11022500	0.13233100	-1.61054300
O	-2.08874400	0.13863300	-2.30498500
C	-0.52221300	-2.47763400	-1.50553600
C	-0.82449600	2.71417500	-0.97044800
O	0.27971900	-3.36901700	-1.31891500
O	-0.22796000	3.60501100	-0.40294500

O	-1.75334900	-2.68700100	-1.98446400
O	-1.94606800	2.90993800	-1.67159500
C	-2.05175300	-4.04486900	-2.30372600
C	-2.40523700	4.25953100	-1.70305000
C	2.13164600	1.35940100	-0.08356800
C	2.63187600	2.57988600	0.30921700
C	3.95110500	2.62892000	0.83610700
C	4.73752800	1.50461400	0.97392800
C	4.24103100	0.22964900	0.58523300
C	2.94532000	0.20240300	0.06454500
C	4.90651700	-1.02375700	0.68627800
C	4.27227100	-2.18105400	0.28698600
C	2.95018600	-2.18851300	-0.23588900
C	2.28644000	-0.98825200	-0.34910800
H	-0.82920900	0.95323600	1.52372900
H	-2.35771600	-1.34353600	0.18532900
H	0.14145500	-1.16348100	1.99373300
H	-0.33839500	-2.64471300	1.15910600
H	-2.82863800	2.25245900	0.60679500
H	-3.52868500	0.73055500	0.04405600
H	-2.39080900	-2.82774400	2.46500100
H	-1.03148200	-2.62650900	3.55720700
H	-4.61254000	1.42012000	2.11919000
H	-3.07763900	1.67130200	2.93130700
H	-2.00399600	-4.66691000	-1.40656600
H	-3.06044400	-4.03426600	-2.71293300
H	-1.34028800	-4.42841500	-3.03789700
H	-3.30992900	4.24731800	-2.30813200
H	-2.62075300	4.61273900	-0.69113700
H	-1.64936200	4.90961600	-2.14906300
H	2.03249700	3.47968700	0.22695100
H	4.34716000	3.59114000	1.14598100
H	5.73928200	1.58741900	1.38742200
H	5.91681200	-1.06485800	1.08494600
H	4.79637700	-3.12781100	0.37535200
H	2.47665300	-3.11582000	-0.53785100
H	-4.46683000	-0.43161300	3.49849100
H	-3.94319700	-1.09856000	1.97474800
H	-2.62976300	-1.22955800	4.49173100
H	-1.64858100	-0.09223900	3.60437700

TS-2$_{Methyl}$-BCN*

C	-1.62568100	-0.62069400	0.40940100
C	-1.62568900	0.62068200	0.40940000
C	-1.99331600	1.89765600	1.05092700
C	-2.81507700	1.60914600	2.32155900

H	-1.08398300	2.46226000	1.29696700
H	-2.57524300	2.51718100	0.35838500
C	-1.99328200	-1.89767200	1.05093500
H	-1.08393800	-2.46225700	1.29697400
H	-2.57520000	-2.51721200	0.35839800
C	-2.81504500	-1.60917300	2.32156800
H	-3.75386700	-1.12429600	2.03058000
H	-3.08604500	-2.56359200	2.79048000
H	-3.08609800	2.56356100	2.79046600
H	-3.75388800	1.12424700	2.03057000
C	-0.24753800	-1.19089000	-1.30164200
C	-0.24754200	1.19089300	-1.30164000
C	0.96476000	0.72172800	-0.84225400
C	0.96476300	-0.72172100	-0.84225500
C	-0.92639700	0.00000100	-1.96964600
O	-1.71616000	-0.00000100	-2.86928400
C	-0.57168500	2.61370000	-1.51182600
C	-0.57167400	-2.61369800	-1.51183300
O	0.13210100	3.53291600	-1.15005100
O	0.13211700	-3.53291200	-1.15006200
O	-1.76029300	-2.78555300	-2.10062600
O	-1.76030400	2.78555100	-2.10062100
C	-2.13725000	-4.14550500	-2.30601200
H	-2.21188600	-4.66845700	-1.34878100
H	-3.10501200	-4.10919500	-2.80317900
H	-1.40010700	-4.65530200	-2.93009000
C	-2.13726900	4.14550200	-2.30600000
H	-3.10503000	4.10919000	-2.80316900
H	-2.21190800	4.66844800	-1.34876700
H	-1.40012800	4.65530700	-2.93007500
C	2.19989800	1.18509700	-0.20452000
C	2.90281300	0.00000600	0.14811700
C	2.75989300	2.40627200	0.09315100
C	4.02582000	2.42657700	0.73927700
H	2.24808900	3.32746700	-0.16090200
C	4.70486200	1.27500600	1.07737500
H	4.47050100	3.38925200	0.97242400
H	5.67087400	1.33706200	1.57180100
C	2.19990300	-1.18508700	-0.20452200
C	2.75990200	-2.40626000	0.09314700
H	2.24810200	-3.32745700	-0.16090800
C	4.02582900	-2.42656100	0.73927300
H	4.47051300	-3.38923500	0.97241900
C	4.14636700	0.00000800	0.78486800
C	4.70486700	-1.27498800	1.07737300
H	5.67087900	-1.33704100	1.57179900

C	-2.07470800	0.75600000	3.32966800
C	-2.82597200	-0.00000800	4.39065000
C	-2.07469200	-0.75600700	3.32967100
H	-1.13223900	1.19622300	3.65206800
H	-3.91077900	-0.00002000	4.31145700
H	-2.45224300	-0.00000200	5.40891500
H	-1.13221400	-1.19620900	3.65207400

Table S3. Energetics (gas phase)

E-Electronic Energy M06-2X/6-311++G(d,p)//M06-2X/6-31G(d)
G-Gibbs Free Energy M06-2X/6-311++G(d,p)//M06-2X/6-31G(d)
ZPE-Zero Point Energy M06-2X/6-31G(d)
H-Enthalpy M06-2X/6-31G(d)
in hartrees

	E	G	ZPE	H
BCN*	-350.010847	-349.85421	0.188468	-349.718324
TCO	-313.17278	-312.997533	0.206436	-312.871855
3	-538.610586	-538.447851	0.200659	-538.244196
TS-3-BCN*	-888.623237	-888.281048	0.389904	-887.964988
TS-3-TCO	-851.780438	-851.41792	0.40887	-851.112754
2_{Methyl}	-1107.228865	-1107.003362	0.270467	-1106.640332
TS-2_{Methyl}-BCN*	-1457.237307	-1456.830452	0.460428	-1456.356821
TS-2_{Methyl}-TCO	-1420.398791	-1419.972114	0.479647	-1419.508398
1	-790.398383	-790.24524	0.192168	-789.995489
TS-1-BCN*	-1140.410297	-1140.074606	0.382312	-1139.714332
TS-1-TCO	-1103.578704	-1103.223662	0.401168	-1102.872194

Table S4. Electronic energies in solvents (SMD, M06-2X/6-311++G(d,p)//M06-2X/6-31G(d))

	1,4-dioxane	methanol	water
BCN*	-350.018712	-350.023487	-350.015553
TCO	-313.178382	-313.180853	-313.172758
3	-538.623038	-538.629224	-538.624072
TS-3-BCN*	-888.641347	-888.65266	-888.641481
TS-3-TCO	-851.796971	-851.807825	-851.796975
2_{Methyl}	-1107.24438	-1107.252262	-1107.245173
TS-2_{Methyl}-BCN*	-1457.258374	-1457.271407	-1457.258282
TS-2_{Methyl}-TCO	-1420.417798	-1420.431123	-1420.418212
1	-790.410576	-790.423688	-790.425296
TS-1-BCN*	-1140.42503	-1140.443626	-1140.439795
TS-1-TCO	-1103.592202	-1103.608711	-1103.604674

NMR

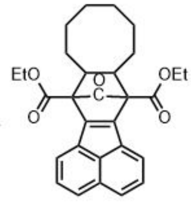

EtO
OEt
O
C
O
O

4

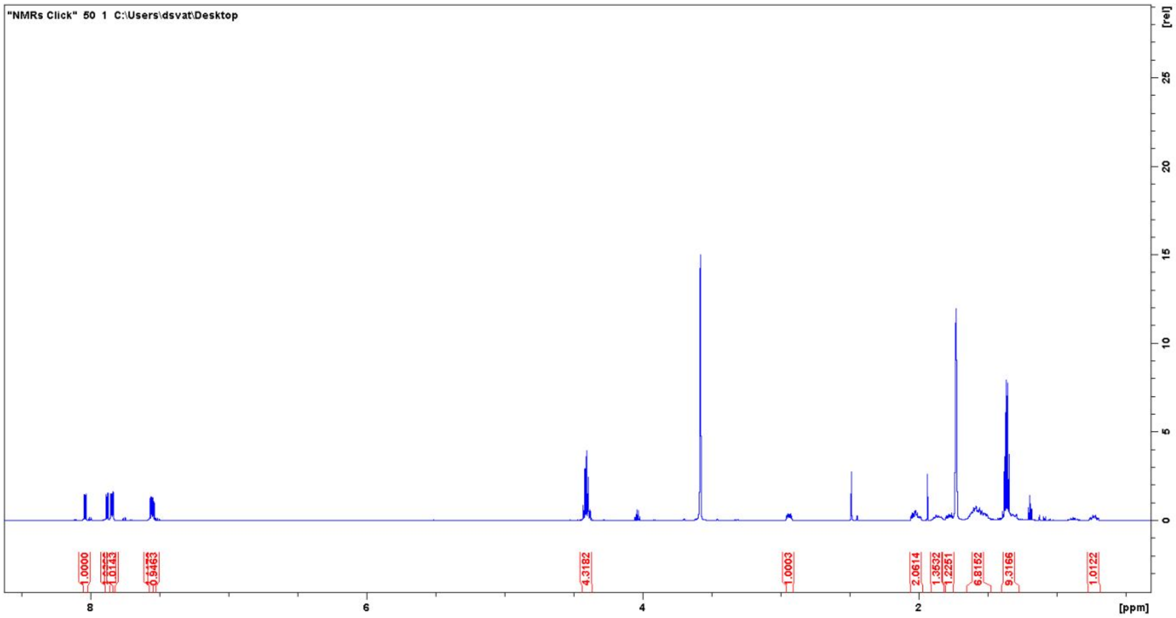

"NMRs Click" 50 1 C:\Users\dsvat\Desktop
[rel]
25
20
15
10
5
0
1.0000
1.0143
0.9463
4.3182
1.0003
2.0614
1.3532
1.2251
6.8152
9.3166
1.0122
8
6
4
2
[ppm]

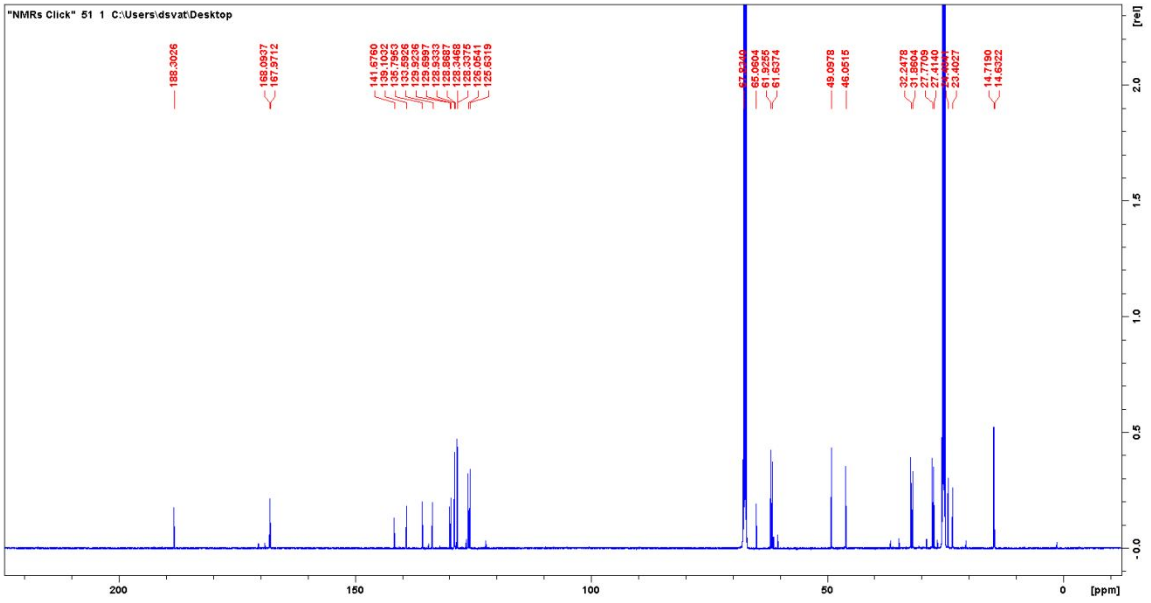

"NMRs Click" 51 1 C:\Users\dsvat\Desktop
[rel]
188.3026
168.0937
167.9712
141.6760
139.1032
135.7953
133.5926
129.9236
129.6997
128.9333
128.8687
128.3468
128.3375
126.0541
125.6319
65.0604
61.9255
61.6374
49.0978
46.0515
32.2478
31.8604
27.7709
27.4140
23.4027
14.7190
14.6322
2.0
1.5
1.0
0.5
0.0
200
150
100
50
0
[ppm]

References

(1) Ding, L.; Ying, H.-Z.; Zhou, Y.; Lei, T.; Pei, J. Polycyclic Imide Derivatives: Synthesis and Effective Tuning of Lowest Unoccupied Molecular Orbital Levels through Molecular Engineering. *Org. Lett.* **2010**, *12* (23), 5522–5525.

(2) Svatunek, D.; Denk, C.; Rosecker, V.; Sohr, B.; Hametner, C.; Allmaier, G.; Fröhlich, J.; Mikula, H. Efficient Low-Cost Preparation of -Cyclooctenes Using a Simplified Flow Setup for Photoisomerization. *Monatsh. Chem.* **2016**, *147*, 579–585.

(3) Denk, C.; Svatunek, D.; Filip, T.; Wanek, T.; Lumpi, D.; Fröhlich, J.; Kuntner, C.; Mikula, H. Development of a18F-Labeled Tetrazine with Favorable Pharmacokinetics for Bioorthogonal PET Imaging. *Angew. Chem. Int. Ed.* **2014**, *53* (36), 9655–9659.

(4) DeForest, C. A.; Tirrell, D. A. A Photoreversible Protein-Patterning Approach for Guiding Stem Cell Fate in Three-Dimensional Gels. *Nat. Mater.* **2015**, *14* (5), 523–531.

(5) Ji, X.; Ji, K.; Chittavong, V.; Aghoghovbia, R. E.; Zhu, M.; Wang, B. Click and Fluoresce: A Bioorthogonally Activated Smart Probe for Wash-Free Fluorescent Labeling of Biomolecules. *J. Org. Chem.* **2017**, *82* (3), 1471–1476.
